# Therapeutic Potential of Bovine Amniotic Membrane in Wound Healing: Insights from a Mouse Model

**DOI:** 10.3390/cells14141040

**Published:** 2025-07-08

**Authors:** Dongwoo Yu, Ye Jin Kwon, Chi Heon Kim, Inbo Han, Jong-Moon Hwang, Kyoung-Tae Kim

**Affiliations:** 1 Department of Neurosurgery, Yeungnam University Hospital, Yeungnam University College of Medicine, Daegu 42415, Republic of Korea; ydwnss@yu.ac.kr; 2 Department of Neurosurgery, Kyungpook National University Hospital, Daegu 41944, Republic of Korea; kyjj0214@naver.com; 3 Department of Neurosurgery, Seoul National University College of Medicine, Seoul 03080, Republic of Korea; chiheon1@snu.ac.kr; 4 Department of Neurosurgery, CHA Bundang Medical Center, CHA University, Seongnam 13496, Republic of Korea; hanib@cha.ac.kr; 5 Department of Rehabilitation Medicine, Happy Rehabilitation Medicine Clinic, Daegu 42122, Republic of Korea; 6 Department of Neurosurgery, Bokwang Hospital, Daegu 42734, Republic of Korea; 7 Joint Institute for Regenerative Medicine, Kyungpook National University Hospital, Daegu 41944, Republic of Korea

**Keywords:** amniotic membrane, wound healings, biologic dressing, skin, mouse

## Abstract

Wound healing involves complex interplay between cellular and molecular events. In this study, we investigated the therapeutic potential of the bovine amniotic membrane (BAM) in wound healing using a mouse model. Twelve male C57BL/6 mice were divided into four groups: negative control (Vehicle), positive control (DuoDERM Extra Thin^®^), amniotic membrane attachment (Amniotic Membrane), and compressed amniotic membrane attachment (Amniotic Membrane with Compression). The dorsal skin of each mouse was excised and wound-healing parameters were assessed over a two-week period. Our results revealed that the Amniotic Membrane and Amniotic Membrane with Compression groups demonstrated significant sustained reductions in the wound area compared to the Vehicle group. These reductions were more pronounced than those observed in the DuoDERM group. Histopathological analysis revealed advanced wound healing characteristics in the BAM-treated groups. Immunohistochemical analysis demonstrated elevated expression levels of wound healing markers (including α-smooth muscle actin, collagen type III, SMAD 1/5/8, and SMAD 2/3) in the BAM-treated groups compared to the control and DuoDERM groups. Conversely, cluster of differentiation 4 levels were significantly lower in BAM-treated groups. Overall, our findings highlight the therapeutic efficacy of BAM and compression in promoting wound healing. Thus, BAM offers a promising therapeutic approach for enhancing wound healing outcomes in clinical settings, potentially by modulating key wound healing pathways and processes.

## 1. Introduction

Wound healing is a complex biological process involving a series of coordinated events aimed at restoring tissue integrity and function [1]. Acute wounds, resulting from trauma, surgery, or other insults, typically undergo timely and efficient healing under normal physiological conditions. However, complications such as infection, inadequate tissue perfusion, or impaired immune responses can impede the healing process, leading to delayed wound closure, chronic wounds, or even tissue loss. Furthermore, full-thickness wounds, characterized by damage extending through the entire dermis into deeper tissues, pose unique challenges to wound healing due to their complex nature and increased risk of complications. Tissue loss, insufficient blood flow, and concurrent medical conditions can result in persistent wounds that pose management challenges [2]. To overcome these challenges and enhance wound healing results, researchers have investigated different therapeutic methods designed to expedite the healing process and encourage tissue regeneration. Skin substitutes are one option for advancing wound care through research, education, and clinical practice. They provide comprehensive wound care services, including advanced wound dressings, surgical interventions, and innovative therapies, to promote optimal healing outcomes. Although skin substitutes are effective in treating various wounds, they also encounter limitations, including resource constraints, limited access to specialized care in underserved regions, and difficulties in implementing evidence-based practices in practical settings.

The amniotic membrane, located closest to the placenta, possesses several distinctive features that make it well-suited for wound healing applications [3]. It is composed of a thin, transparent matrix rich in growth factors, cytokines, and extracellular matrix components, which promote cell proliferation, migration, and tissue regeneration [4]. Its natural biocompatibility and lack of immunogenicity make it an ideal candidate for use as a skin substitute in various wound types, including burns, chronic ulcers, and surgical wounds. Additionally, the amniotic membrane can serve as a scaffold for cell attachment, proliferation, and differentiation, further enhancing its potential as a regenerative therapy for tissue repair [5]. These characteristics have made the human amniotic membrane (hAM) a valuable biological material for various dressing applications. The use of hAM in surgery dates back to 1910, when Davis first reported its application in skin transplantation [6]. Since then, hAM has been widely studied for its anti-inflammatory, anti-fibrotic, and pro-regenerative properties. Notably, Mermet et al. demonstrated the effectiveness of hAM in promoting healing in chronic leg ulcers, supporting its broader use in regenerative medicine [7]. However, its clinical use is limited by factors such as donor availability, ethical concerns, cost, and variability in quality. Although structural variations exist between species, bovine amniotic membrane (BAM) and hAM share key histological characteristics, including a collagen-rich stromal matrix and anti-inflammatory properties, supporting their functional similarity [8].

In this study, we explore the therapeutic benefits of BAM in wound healing using a mouse model. By conducting a thorough assessment of wound healing parameters, such as tissue regeneration, inflammation, and collagen deposition, our goal is to understand the effectiveness and underlying mechanisms of amniotic membrane in facilitating effective wound repair. This study aims to advance the development of novel therapeutic approaches for addressing challenges in wound healing within clinical practice, utilizing the distinct properties of amniotic membrane as a regenerative biomaterial.

## 2. Materials and Methods

### 2.1. Animal Preparation

Twelve male C57BL/6 mice (7 weeks old, weighing 23–25 g) were recruited. They were provided with a standard rodent diet and housed in an animal facility (maintained at 20–25 °C with 12 h light cycles) following institutional guidelines for one week before the experiment.

We induced general anesthesia in the C57BL/6 mice using 100% oxygen at a flow rate of 1 L/min and 5% isoflurane. The anesthesia was then maintained at 0.5 to 2% isoflurane. We removed dorsal skin fur with an electric clipper and applied hair removal cream to the back area for 3 min, ensuring it did not exceed 5 min to prevent skin damage. Subsequently, the skin was cleaned with wet gauze, and each mouse was housed individually to minimize scratching and chewing.

**Figure 1:**
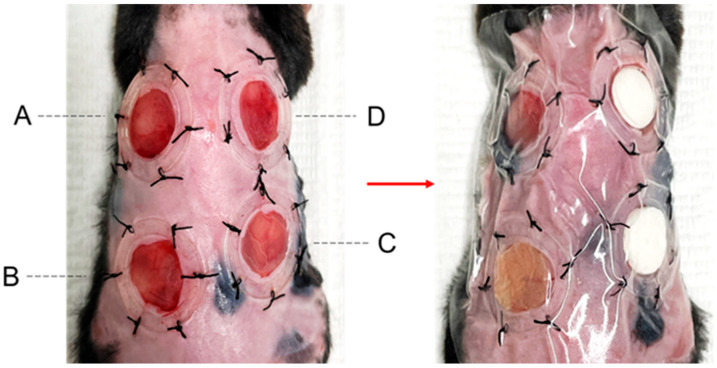
Experimental setup for wound creation and treatment categorization. The excisional wounds were marked using an 8 mm biopsy punch, followed by the placement of a silicone splint with an inner diameter of 10 mm to ensure centralized incision. Each mouse underwent the procedure, resulting in four distinct wound types: negative control (**A**, Vehicle), positive control (**B**, DuoDERM Extra Thin^®^), amniotic membrane attachment (**C**, Amniotic Membrane), and compressed amniotic membrane attachment (**D**, Amniotic Membrane with Compression).

We marked the location of the excisional wound using an 8 mm biopsy punch and proceeded to make an incision. Additionally, a silicone splint with an inner diameter of 10 mm was sutured and fixed, ensuring the incision was positioned at the center. This entire process was repeated to create a total of four wounds per mouse, categorized as follows: negative control (Group A, Vehicle), positive control (Group B, DuoDERM Extra Thin^®^), amniotic membrane attachment (Group C, Amniotic Membrane), and compressed amniotic membrane attachment (Group D, Amniotic Membrane with Compression) (Figure 1). In the Amniotic Membrane with Compression group, compression was applied to enhance contact between the membrane and the wound surface. A sterile plastic disk (10 mm in diameter, 5 mm in thickness) was placed directly over the wound after BAM application, and the disk was secured using a transparent film dressing (Tegaderm™, 3M, St. Paul, MN, USA).

For the subsequent two weeks, we applied materials daily to the backs of both the positive control and test groups. Tegaderm was used across the entire back to prevent the wound from drying out and to protect against the mice tearing the splint. The recovery process involved placing the mice back into their individual cages in a warm environment until fully recovered. Each day, following anesthesia, photographs were taken, and test substances were applied to the back skin of each group of experimental mice. The wound area was then calculated using Image J image analysis software, and the recovery degree was determined as the ratio of the wound area on day 0 to the initial wound size.

### 2.2. Bovine Amniotic Membrane Preparation

The BAM was isolated from the placentas of cattle managed under livestock and zoonotic infectious disease control protocols. The epithelial layer was removed, and the tissue was treated with 70% ethanol for 150 *s*, followed by three washes with phosphate-buffered saline (PBS). The membrane was then neutralized to a pH of 6.5–7.0. To eliminate immunogenic cells from the remaining stromal layer, the tissue was treated with a decellularization solution containing 0.25% (*w*/*v*) trypsin, 0.02% (*w*/*v*) EDTA, and 0.9% (*w*/*v*) NaCl at pH 7.0 for 60 min. After decellularization, the membrane was thoroughly rinsed with sterile ultrapure water to remove residual reagents. The resulting acellular bovine amniotic membrane was freeze-dried into a thin film to facilitate storage and direct application to wound sites. For final preparation, the freeze-dried membrane was sealed in sterile packaging and sterilized using 25 kGy gamma irradiation.

### 2.3. Wound Area Measurement

We measured the area of inner and outer ring using Image J. We calculated the total area of contraction as follows: area of outer ring at day 0—area of outer ring at day t (TA_0_ − TA_t_); wound area of contraction: the ratio of wound area (WA) to total area (TA) * total area of contraction [(WA_0_/TA_0_) * (TA_0_ − TA_t_)]. We designed the diameter of the inner ring to be 10 mm, and the outer ring to be 14 mm. A combination of wound epithelialization and wound contraction leads to wound reduction [area of inner ring at day 0—area of inner ring at day t (WA_0_ − WA_t_)]. Therefore, wound epithelialization area can be calculated as follows: area of wound reduction − wound area of contraction [(WA_0_ − WA_t_) − (TA_0_ − TA_t_) * (WA_0_/TA_0_)]. The rate of wound epithelialization was calculated as the proportion of the wound epithelialization area compared to the total wound reduction area [9].

### 2.4. Histopathological Evaluation

We harvested wound tissue from all groups at the endpoint of our research. The wound tissue was fixed using 4% paraformaldehyde. After fixation, the tissues were embedded in paraffin, sectioned at 4 µm thickness, and stained with hematoxylin and eosin (H&E). Subsequently, photographs of the H&E-stained sections were captured using a microscope. For histological comparison, normal (unwounded) skin tissue adjacent to the surgical site was collected from the same animals and used as a control. The control samples underwent the same fixation, embedding, sectioning, and staining procedures as the experimental wound tissues.

Formalin-fixed tissue samples were embedded in paraffin and sectioned to a thickness of 4 μm. Tissue sections were deparaffinized in xylene and rehydrated through alcohol solutions. Sections were stained using the Masson’s Trichrome staining kit following the manufacturer’s protocol. Tissue sections were immersed in Weigert’s Iron Hematoxylin for 10 min, staining nuclei black-blue. Sections were then stained with Biebrich Scarlet–Acid Fuchsin for 15 min, resulting in muscle fibers appearing red and cytoplasm showing varying shades of pink. Differentiation was achieved using a phosphomolybdic–phosphotungstic acid solution for 10 min. Collagen fibers were stained blue with Aniline Blue Solution for 5 min. Dehydration was performed through graded alcohols, and sections were mounted using a permanent mounting medium. Stained tissue sections were observed under a light microscope, and images were captured using appropriate filters.

Thin tissue samples mounted on slides were permeabilized with 0.1% Triton X-100 for 10 min. To prevent non-specific antibody binding, the cells were blocked for 1 h using a solution containing 3% bovine serum albumin (BSA). Samples were incubated with antibodies (1:200 dilution) overnight at 4 °C to allow for specific binding. Unbound primary antibodies were washed with phosphate-buffered saline (PBS). Fluorescently labeled secondary antibodies (1:200 dilution) were applied for 1 h at room temperature. Unbound secondary antibodies were removed by washing with PBS. After staining with mounting solution containing 4′,6-diamidino-2-phenylindole (DAPI), the samples were observed under a microscope. Immunofluorescence staining analysis was performed using ImageJ 1.54f software, and results were quantified as the ratio of marker-positive cells to DAPI-positive nuclei in randomly selected microscopic fields.

### 2.5. Statistical Analysis

All data are presented herein as the mean ± standard deviation (SD). A statistical analysis to compare the mean values of the two groups was performed using the Mann–Whitney U test, a nonparametric test, as the sample size was small and it cannot be assumed as a normal distribution. Statistical significance was set at *p* < 0.05. Statistical analyses were performed using the Prism software (version 8.0; GraphPad Software, La Jolla, CA, USA).

## 3. Results

### 3.1. Wound Area

**Figure 2:**
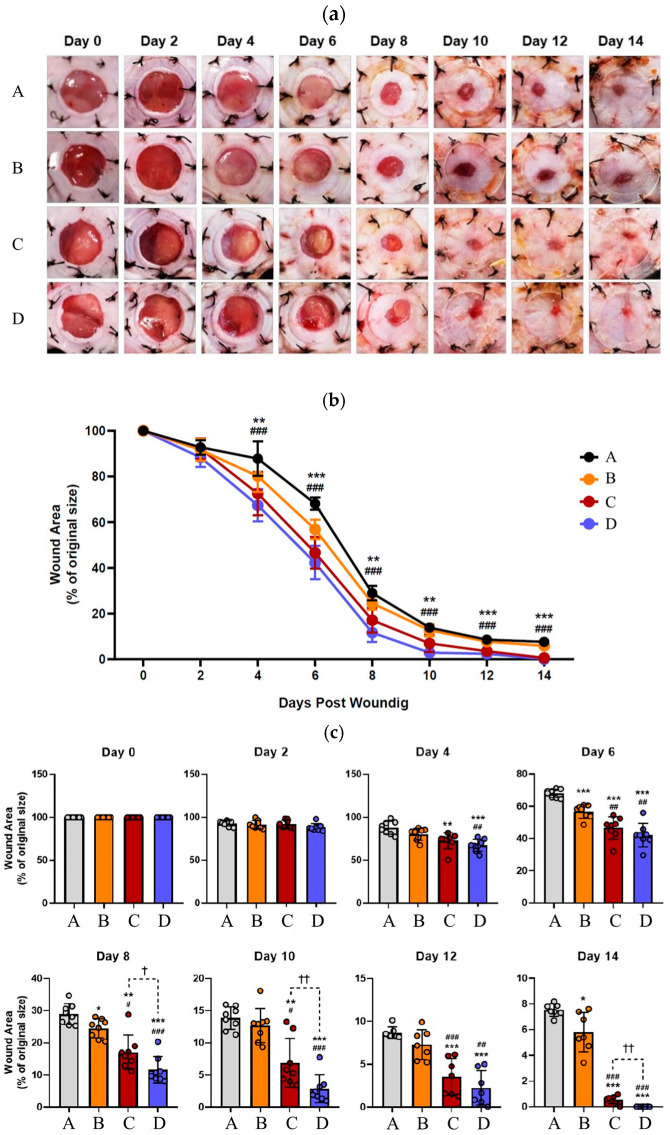
(**a**) Representative images depict dorsal skin wounds in excisional splinting mice following 14 days of continuous treatment with specified compounds. (**b**) The wound area (% of original size) of wound healing in excisional splinting mice after continuous treatment with indicated compounds for 14 days is presented. Data are expressed as the mean ± SD. Statistical significance was determined as follows: ** = *p* < 0.01, and ***, ### = *p* < 0.001 (*: Vehicle vs. Amniotic membrane, #: Vehicle vs. Amniotic Membrane with Compression). (**c**) The wound area (% of original size) every two days of wound healing. Data are expressed as the mean ± SD. Statistical significance was determined as follows: *, #, † = *p* < 0.05, **, ##, †† = *p* < 0.01, and ***, ### = *p* < 0.001 (*: versus Vehicle, #: versus DuoDERM, †: versus Amniotic Membrane). Treatment groups are indicated as follows: (A) negative control (Vehicle), (B) positive control (DuoDERM Extra Thin^®^), (C) amniotic membrane attachment, and (D) compressed amniotic membrane attachment.

The evaluation of wound areas revealed statistically significant reductions in the DuoDERM group compared to the Vehicle group on days 6, 8, and 14. Similarly, both the Amniotic Membrane and Amniotic Membrane with Compression groups exhibited significant decreases in the wound area compared to the Vehicle group after Day 4. Furthermore, they continued to show statistically significant reductions compared to the DuoDERM group after Day 6. Notably, particularly after day 8, the Amniotic Membrane with Compression group demonstrated a more pronounced reduction than the Amniotic Membrane group (Figure 2).

### 3.2. Histopathological Analysis

**Figure 3:**
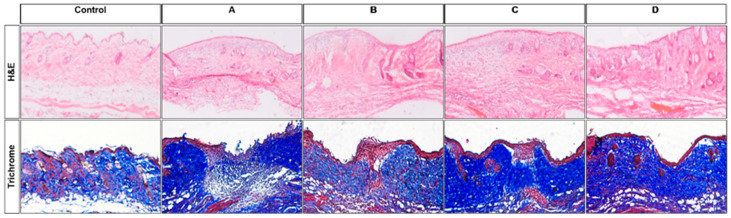
H&E staining reveals general tissue morphology across the five groups. The amniotic membrane and amniotic membrane with compression groups exhibit characteristics indicative of more advanced wound healing, including organized epidermal and dermal layers, as well as fewer inflammatory cells. Trichrome staining depicts collagen deposition and organization in the wound area. The Amniotic Membrane and Amniotic Membrane with Compression groups demonstrate a more structured and organized collagen network, suggesting enhanced tissue strength and integrity during the wound healing process.

H&E-stained sections were used to assess and compare the overall structure and composition of the wounds. The Amniotic Membrane and Amniotic Membrane with Compression groups exhibited characteristics suggestive of advanced wound healing such as organized epidermal and dermal layers and reduced inflammatory cell infiltration. Trichrome staining revealed collagen deposition and organization in the wound. Both the Amniotic Membrane and Amniotic Membrane with Compression groups exhibited a more structured and organized collagen network, suggesting enhanced tissue strength and integrity during the wound healing process (Figure 3).

**Figure 4:**
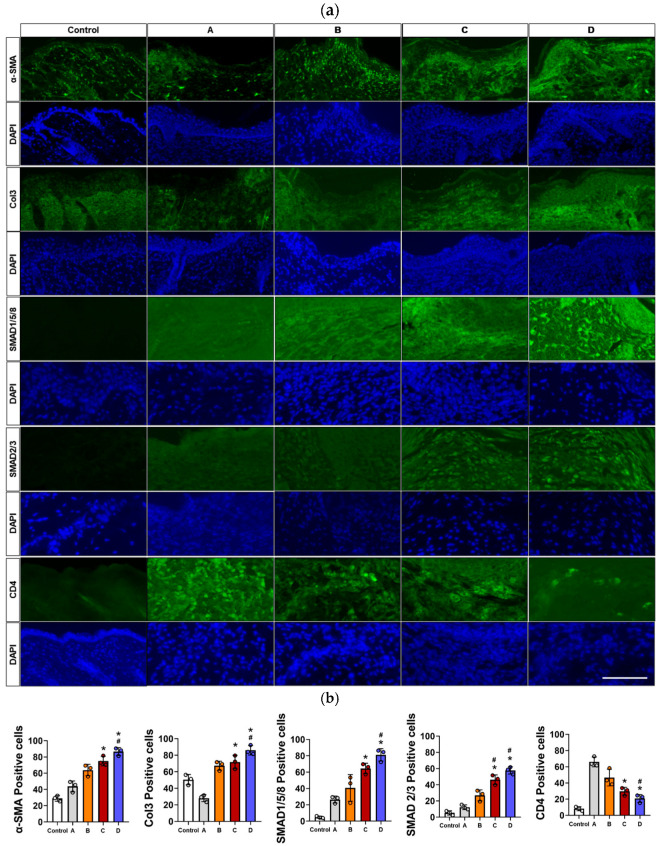
(**a**) Immunofluorescence staining analysis revealed higher expression levels of α-SMA, Col 3, SMAD 1/5/8, and SMAD 2/3 in the Amniotic Membrane and Amniotic Membrane with Compression groups compared to the Control, Vehicle, and DuoDERM groups. Immunohistochemical results demonstrated a decrease in CD4 levels in the Amniotic Membrane groups, suggesting an immunomodulatory effect exerted by the treatment. All microscopic images are accompanied by a scale bar representing 200 μm. (**b**) Data are expressed as the mean ± SD. Statistical significance was determined as follows: *, #, = *p* < 0.05, (*: versus Vehicle, #: versus DuoDERM).

In immunohistochemical analysis of wound healing markers, elevated expression levels of α-smooth muscle actin (α-SMA), collagen type III (Col 3), SMAD 1/5/8, and SMAD 2/3 were observed in the Amniotic Membrane and Amniotic Membrane with Compression groups compared to the Control, Vehicle, and DuoDERM groups. Cluster of differentiation 4 (CD 4) levels in the Amniotic Membrane and Amniotic Membrane with Compression groups were significantly lower than those in the Vehicle and DuoDERM groups based on immunohistochemical quantitative analysis (Figure 4). A comparative assessment of Col 1 and CD 68 levels revealed no significant differences across groups.

## 4. Discussion

AM possesses a unique set of characteristics that make it an attractive candidate for therapeutic applications in wound healing. BAM offers several practical advantages over hAM, including greater availability, lower cost, and fewer ethical or cultural concerns. BAM may also provide superior mechanical properties, such as higher tensile strength and water absorption [10]. Although the risk of zoonotic transmission (e.g., bovine spongiform encephalopathy) exists, it can be minimized through decellularization and appropriate processing protocols [11]. Derived from the innermost layer of the fetal membranes that encloses the amniotic cavity, AM exhibits remarkable biocompatibility, low immunogenicity, and favorable mechanical properties, making it an ideal scaffold for tissue regeneration without causing harm to the human body [12]. The natural composition of AM comprises a rich extracellular matrix (ECM) containing collagen III, hyaluronic acid, and growth factors, which provide a conducive microenvironment for cellular attachment, proliferation, and differentiation [13,14]. Additionally, AM exhibits anti-inflammatory and anti-microbial properties, attributed to the presence of bioactive molecules such as cytokines, chemokines, and protease inhibitors, which modulate immune responses and mitigate microbial colonization [13,15,16,17]. A previous study demonstrated the wound healing potential of solubilized bovine amniotic membrane extract (AME) in both in vitro and in vivo models. AME significantly enhanced fibroblast migration in a scratch assay and promoted re-epithelialization and organized dermal regeneration in a rabbit ear wound model, without inducing hyperproliferation or scarring [18]. These results support the biological activity of BAM-derived materials in tissue repair. Our study complements these findings by using intact, decellularized BAM in a murine excisional wound model, further demonstrating enhanced healing through wound area reduction and histopathological improvements, including increased collagen deposition and reduced inflammation. These results support the therapeutic potential of BAM in promoting wound healing. The observed effects may be attributed to BAM’s anti-inflammatory, anti-microbial, and pro-regenerative properties, which help create a favorable microenvironment for tissue repair.

In this study, both the Amniotic Membrane group and the Amniotic Membrane with Compression group showed significant reductions in wound area compared to the Vehicle group and the DuoDERM group. The amniotic membrane contains three separate layers, namely the epithelial layer, the basement membrane, and the mesenchymal layer. Within the epithelial layer lies a single layer of epithelial cells, while the basement membrane is characterized by its delicate composition of reticular fibers that connect with the epithelial layer through interdigitation. As for the mesenchymal layer, it is defined by an avascular collagen matrix, which is further divided into compact, fibroblast, and sponge layers [19]. While the amniotic membrane exhibits structural variations, its histological composition closely resembles that of the skin, characterized by a multilayered epithelium and basement membrane. This similarity provides several advantages in wound healing, including enhanced epithelialization, decreased inflammatory responses, and suppression of scar formation and bacterial proliferation [20,21]. The amniotic membrane shields skin wounds from external factors, reduces the leakage of water, electrolytes, protein, and energy, alleviates discomfort, addresses the shortcomings of current dermal substitutes susceptible to infections, and fosters wound healing via the activation of diverse growth factors [22]. In our research, the group subjected to compression using the amniotic membrane exhibited the most favorable outcomes. Compression assists by raising tissue pressure, thereby decreasing edema and enhancing blood circulation to the wound area. This improved blood flow facilitates the delivery of more oxygen and nutrients to the wound site, which promotes faster healing [23,24]. Beidler et al. [25] reported that compression therapy downregulates levels of pro-inflammatory cytokines and metalloproteinases while boosting levels of anti-inflammatory cytokines. Due to these mechanisms, compression may aid in reducing scar formation and enhancing the overall cosmetic appearance of a healed wound.

The transforming growth factor-beta (TGF-β) pathway plays a critical role in the process of wound healing, particularly in the phases of inflammation, proliferation, and remodeling. The SMAD proteins are key intracellular mediators of the TGF-β signaling pathway [26]. In our study, we confirmed an increase in SMAD1/5/8 and SMAD2/3 levels not only within the Amniotic Membrane group but also within the Amniotic Membrane and Amniotic Membrane with Compression groups. In particular, SMAD2/3 are receptor-regulated SMADs primarily responsible for mediating signaling from TGF-β ligands [27]. An elevation of SMAD2/3 in the wound healing process indicates increased activation of the TGF-β signaling pathway, which contributes to the regulation of various cellular processes essential for effective wound repair. It promotes the proliferation and migration of various cell types involved in wound healing, including fibroblasts, endothelial cells, and keratinocytes [26].

Col3 also plays a significant role in wound healing, particularly during the early stages of the process [28]. Throughout the inflammatory and proliferative stages of wound healing, fibroblasts predominantly synthesize Col3, a key collagen [29]. The elevation of Col3 in the wound healing process typically indicates the early stages of wound repair, particularly during the proliferative phase. The observed elevation of Col3 in the group treated with BAM indicates a significant enhancement in early wound healing processes. Collagen is a structural protein that provides strength and support to tissues. Collagen enhances wound healing through its strong affinity to soft tissue and blood vessels, facilitating the replacement of host cells and vessels through accelerated penetration into the scaffold [30]. In trichrome staining, a more structured and robust collagen network was confirmed in the group treated with BAM. This organized collagen network is important for tissue strength and integrity during wound healing.

α-SMA is a marker protein for myofibroblasts, specialized contractile cells that play a central role in wound contraction. It significantly influences wound healing by enhancing myofibroblast differentiation and contractile function [31]. In our study, we observed an increase in α-SMA expression in the group treated with BAM compared to the control group, indicating its potential to enhance the activity of myofibroblasts. Myofibroblasts contribute to wound contraction and scar formation, potentially expediting the observed faster wound closure. Myofibroblasts exert contractile forces by assembling actin stress fibers containing α-SMA. These forces enable myofibroblasts to draw the wound edges closer together, thus facilitating wound closure [32]. In addition to their contractile function, myofibroblasts also contribute to extracellular matrix remodeling during wound healing. They produce and organize collagen fibers and other extracellular matrix components, facilitating the restructuring and maturation of the wound tissue [33].

BAM contains bioactive molecules and growth factors that can modulate the inflammatory response [34]. CD4 counts and inflammation state are often interrelated, reflecting the complex interactions between the immune system and inflammatory processes in various disease states. In our study, immunohistochemical results showed a decrease in CD4 levels in the BAM-treated groups, suggesting an immunomodulatory effect exerted by the treatment. Several studies reported that amniotic membrane can modulate the production and activity of pro-inflammatory cytokines such as tumor necrosis factor-alpha TNF-α, interleukin-1 beta, and interleukin-6 [35,36,37]. Additionally, amniotic membrane harbors numerous anti-inflammatory cytokines and growth factors, which can diminish the expression of pro-inflammatory cytokines while fostering tissue regeneration [38,39]. These properties contribute to amniotic membrane’s effectiveness in promoting wound healing and tissue regeneration.

Our study highlights BAM’s efficacy in promoting wound healing compared to control groups. This improved wound healing is attributed to multiple mechanisms. Firstly, BAM triggers the TGF-β signaling pathway, boosting collagen production and facilitating extracellular matrix remodeling crucial for tissue repair. Secondly, BAM augments myofibroblast activity, facilitating wound contraction and tissue regeneration. Additionally, BAM demonstrates anti-inflammatory effects by suppressing pro-inflammatory cytokine expression and modulating immune cell function, creating a conducive environment for wound healing. These findings emphasize BAM’s potential as a therapeutic option for enhancing efficient wound healing and tissue regeneration in clinical settings. Although rapid wound closure and reduced inflammation are beneficial, increased myofibroblast activity and active TGF-β signaling also raise concerns regarding potential fibrotic responses. Careful monitoring for excessive fibrosis or scar formation in the long term is essential.

This study was conducted in a mouse model with normal wound healing capacity. While the results are promising, caution is warranted when extrapolating these findings to chronic or impaired wounds in humans. Further research using chronic wound models is necessary to evaluate the clinical applicability of BAM in such contexts.

## 5. Conclusions

The results of our study demonstrated that BAM facilitates efficient wound closure and tissue regeneration through multiple mechanisms. These findings underscore the potential of BAM as a valuable therapeutic option for enhancing wound healing outcomes in clinical settings. Nevertheless, careful consideration of potential fibrotic responses and long-term monitoring of excessive fibrosis or scar formation are warranted. Overall, these findings advance our understanding of the role of BAM in wound healing and highlight its potential applications in clinical practice.

## Data Availability

The datasets generated and/or analyzed during the current study are available from the corresponding author on reasonable request.

## Abbreviations

The following abbreviations are used in this manuscript:

hAM	human amniotic membrane
BAM	bovine amniotic membrane
IACUC	Institutional Animal Care and Use Committee
PBS	phosphate-buffered saline
SD	standard deviation
DAPI	4′,6-diamidino-2-phenylindole
αSMA	α-smooth muscle actin
CD4	cluster of differentiation 4
ECM	extracellular matrix
TGF-β	transforming growth factor-beta
TNF-α	tumor necrosis factor-alpha
